# Rendezvous Stenting Technique for Anastomotic Leak After Total Gastrectomy: A Feasibility Study

**DOI:** 10.3390/medicina62020352

**Published:** 2026-02-10

**Authors:** Konstantinos Saliaris, Sofia Katsila, Tania Triantafyllou, Eleni Kitsou, Konstantinos Kakounis, Panagiotis Varsos, Alexandra Triantafyllou, Andreas Theodorou, Athanasios G. Pantelis, Vassiliki Xiromeritou, Dimitrios Theodorou

**Affiliations:** 1 1st Propaedeutic Surgery Clinic, Hippocration General Hospital, 114 Vass. Sofias Ave., 11527 Athens, Greece; saliarisk@med.uoa.gr (K.S.); elenimedkitsou@gmail.com (E.K.); pvarsos@yahoo.gr (P.V.); alexandra_30fyl@hotmail.com (A.T.); antheo300@gmail.com (A.T.); ath.pantelis@gmail.com (A.G.P.); dimitheod@netscape.net (D.T.); 2 Department of Gastroenterology, Hippocration General Hospital, 114 Vass. Sofias Ave., 11527 Athens, Greece; sophie_katsila@hotmail.com (S.K.); kostas.kakounis@gmail.com (K.K.); xiromeritou.vasiliki.md@gmail.com (V.X.)

**Keywords:** gastrectomy, stent, anastomotic leak

## Abstract

*Background and Objectives*: Anastomotic leak following total gastrectomy and Roux-en-Y reconstruction remains a challenging and potentially morbid clinical scenario. Systemic support and resuscitation with simultaneous local sepsis control remain pillars of treatment. The therapeutic strategy may vary among different centers depending on the severity of clinical presentation, the degree of contamination and the hospital resources. The aim of this study is to introduce the rendezvous stenting technique, which combines washout of the abdominal cavity and endoscopic stenting under direct vision in selected patients who require reoperation. *Materials and Methods*: A retrospective descriptive analysis of severely ill patients suffering an anastomotic leak from an esophagojejunal anastomosis, who had been operated on in our department during the last five years was performed. Patient demographics, perioperative data and surgical outcomes were collected. *Results*: Since 2018, six anastomotic leak patients underwent stenting of anastomotic leak using the rendezvous technique during reoperation. Stenting was effective in controlling local contamination in five out of six patients (83.3%). One patient required repeat stent placement due to improper stent width. *Conclusions*: Anastomotic stenting using the rendezvous technique is a safe and feasible technique. Combining drainage of the abdominal cavity and stent fixation allows for control of the contaminated field as well as minimizing the risk of stent migration.

## 1. Introduction

Anastomotic leak is a dreadful complication of upper gastrointestinal tract surgery as it is accompanied by significant morbidity and mortality [1,2,3,4]. Despite advances in imaging modalities allowing for early recognition and grading of an anastomotic leak, optimal treatment strategy in not clear in all cases [5,6]. Technical advances in modern treatment provide a plethora of tools for control of local contamination [3]. However, even in high-volume centers, esophagojejunal anastomotic leak remains a complication associated with dismal overall prognosis, extended hospital stays, and high in-hospital mortality rates (estimated 7–10% mortality rate) [2,3,4,5]. Even in cases of appropriate anastomotic leak treatment, surviving patients face decreased overall survival, worse oncological outcomes, and poorer quality of life, owing to inability to receive adjuvant treatment and overall frail status [4,5,6].

Management of upper GI anastomotic leaks can be complex, with strategies ranging from non-invasive measures to reoperation depending on the patient’s clinical condition, comorbidities, the nature of disease, and local resources and expertise. Initial treatment strategy focuses on patient resuscitation and optimal supportive care. Simultaneously, measures to address local contamination are employed as needed [3,6]. These range from endoscopic methods to reoperation for diversion or reconstruction of the GI tract [6,7]. Reoperation for sepsis control is reserved for severely septic patients who do not respond to initial resuscitative support and consequently require emergency laparotomy for washout and drainage of intra-abdominal or intrathoracic fluid collections [6].

Endoscopic procedures include clip or stent placement, endoscopic negative pressure therapy (EndoVac) or even combination of the aforementioned techniques, with modern negative pressure stents [6,7]. Endoscopic placement of a stent through the anastomosis is a popular technique, either alone or in conjugation with other modalities, in many high-volume centers [5,6,7,8,9,10]. The basic idea behind its use is that the fully covered stent contains the septic factor within the gastrointestinal tract lumen acting as a seal of the anastomotic defect, avoiding the need for reconstruction or diversion of the gastrointestinal tract [10]. The stent provides a scaffold for tissue growth and healing of the mucosal defect [10].

Stenting over the anastomosis remains a popular modality among upper GI surgeons, as it allows for oral diet continuation preventing the need for artificial feeding, namely parenteral nutrition, feeding jejunostomy, etc., while avoiding frequent endoscopic procedures [10,11,12,13]. Various stenting methods have been utilized over the years depending on patient factors, hospital resources and operator preference and expertise [10,11,12,13]. Despite improvements in stenting technology, stent displacement and migration remains a major, potentially fatal complication that hinders their effectiveness and popularity among endoscopists [11,12,13,14]. Stent migration rates may be as high as 32.1% and are described as the main driver of stent-related complications in anastomotic leak patients [15]. Endoscopic vacuum therapy has gained popularity among endoscopists and clinicians alike, owing to its high success rate of containing local septic factors and rapid anastomotic defect healing [11]. However, frequent endoscopic application is required and oral feeding is not feasible, rendering the technique suboptimal [11].

Migration is the major complication of stenting over the anastomotic defect, preventing the technique’s wide implementation. Stenting technique modifications, including endoscopic stent anchoring and fully covered stents, although reducing migration rates, have not proven fully successful in preventing stent dislodgement [16]. In order to address stent migration, a modified rendezvous stent placement technique has been utilized in our department during the last 3 years. The rendezvous technique in surgery refers to a combined antegrade and retrograde approach—often integrating surgical, endoscopic, and percutaneous methods—to achieve successful ductal access and facilitate stenting when single-modality access fails, commonly employed in hepatobiliary surgery, in order to improve biliary cannulation success [17]. Selected septic patients with esophagojejunal anastomotic leak were subjected to reoperation with simultaneous intraoperative stent placement and surgical fixation. Full-thickness suture anchoring of the stent can provide enough structural integrity to confine the stent’s movement. In the present study we present the technique and our initial experience using it in a short case series of patients suffering anastomotic failure after total gastrectomy.

## 2. Materials and Methods

The intraoperative rendezvous stenting technique has been implemented in our department over the last three years as part of a standardized surgical strategy for the management of severe esophagojejunal anastomotic leakage following total gastrectomy. This retrospective case series includes consecutive patients who underwent total gastrectomy with Roux-en-Y reconstruction in our department and subsequently developed a postoperative esophagojejunal anastomotic leak.

All patients included in the study presented with a radiologically confirmed anastomotic leak, diagnosed by contrast-enhanced computed tomography (CT), within the first 15 postoperative days. In all cases, the anastomotic leak was complicated by intra-abdominal sepsis leading to multiorgan failure, necessitating urgent surgical intervention. Sepsis was defined and diagnosed according to the 2021 Surviving Sepsis Campaign Guidelines. Given the severity of the clinical condition, all patients were managed operatively, and non-operative or purely endoscopic treatment strategies were not considered appropriate. All surgical procedures, including the initial gastrectomy and subsequent reoperations, were performed by the same specialized upper gastrointestinal surgical team, ensuring homogeneity in operative technique and perioperative decision-making. Patients who were deemed suitable candidates for relaparotomy underwent surgical exploration with thorough peritoneal lavage and establishment of wide and effective drainage of intra-abdominal collections. During the same operative session, intraoperative rendezvous stenting was performed.

The rendezvous technique consisted of intraoperative placement of an esophageal self-expanding covered stent under direct surgical guidance, facilitating precise positioning across the esophagojejunal anastomosis. Stent deployment was performed in collaboration with endoscopic guidance when required, and fixation was achieved intraoperatively to minimize the risk of migration. The primary aim of stent placement was to control ongoing leakage, protect the anastomosis, and allow sepsis control in conjunction with surgical source control. Demographic, clinical, and perioperative data were collected from institutional medical records. All patients or their legally authorized representatives provided informed consent prior to both the initial operation and the subsequent reintervention. The study was conducted in accordance with institutional ethical standards and the principles of the Declaration of Helsinki.

Inclusion Criteria

- CT-proven esophagojejunal anastomotic leakage occurring within 15 days after total gastrectomy with Roux-en-Y reconstruction
- Presence of sepsis, as defined by the 2021 Surviving Sepsis Campaign Guidelines
- Written informed consent obtained from the patient or next-of-kin

Exclusion Criteria

- Patients managed with non-operative or conservative treatment strategies

### 2.1. Technical Description

The rendezvous stenting technique takes place in the operating room. After appropriate resuscitation and supportive measures, patients are taken to theater. The peritoneal cavity is accessed through a standard supraumbilical midline incision and appropriate washing and drainage of intra-abdominal collections is performed. Depending on preoperative imaging, chest tubes may be inserted as needed. Following that, an upper GI endoscopy is performed in standard fashion. The endoscopist’s first goal is accurate anastomotic leak site evaluation, taking the mucosal defect size, the quality of surrounding tissue and the presence of foreign bodies into consideration. After endoscopic washing and debris suction, the most suitable stent size is selected. All stents used in our case series were straight esophageal stents. Choice of stent size is subjective, and the decision is left to the discretion of the endoscopy team. Stent size is elected aiming to cover the anastomotic defect while anchoring on healthy tissue. In our series only fully covered self-expanding metal stents (FC-SEMS) in two sizes were used (10–12 cm length and 20–25 mm diameter) in order to achieve sufficient anastomosis sealing.

**Figure 1:**
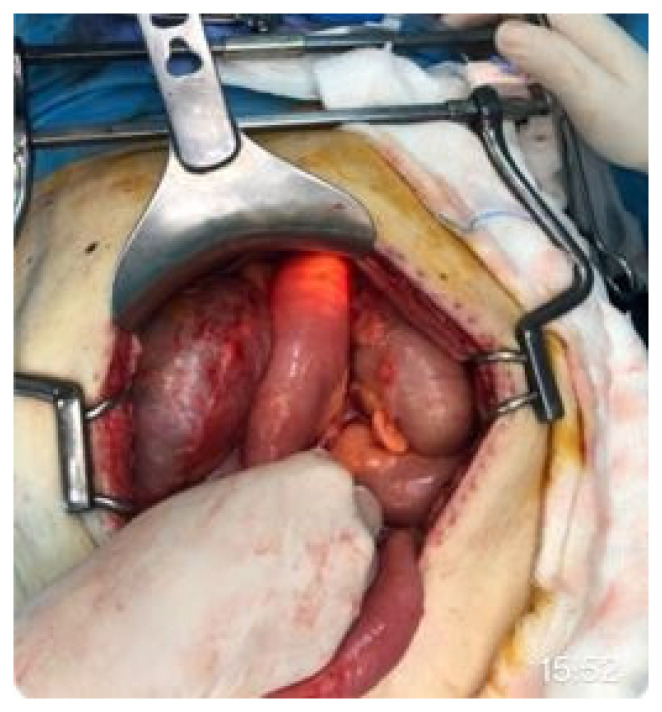
Intraoperative visualization of the endoscope entering the jejunal loop. Manual confirmation of distal landmarks allows for accurate stent deployment.

**Figure 2:**
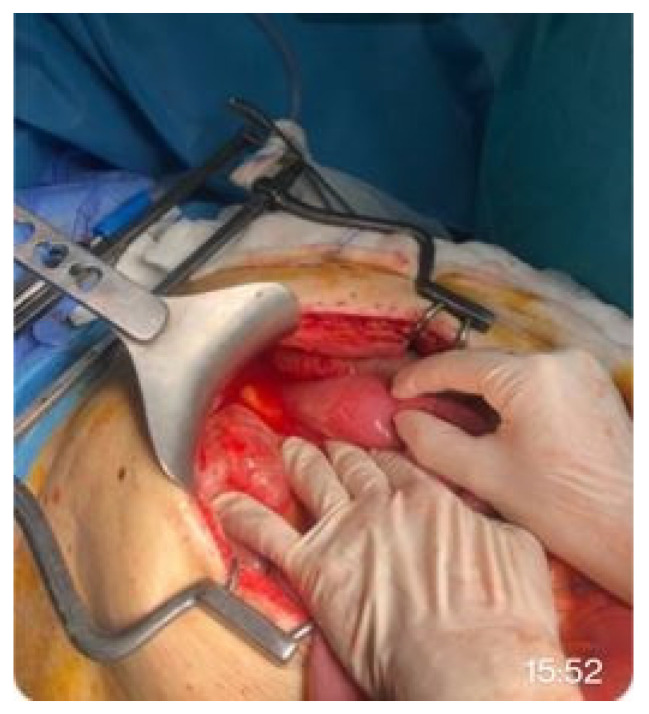
Manual assistance and fixation for proper stent placement over the anastomosis.

**Figure 3:**
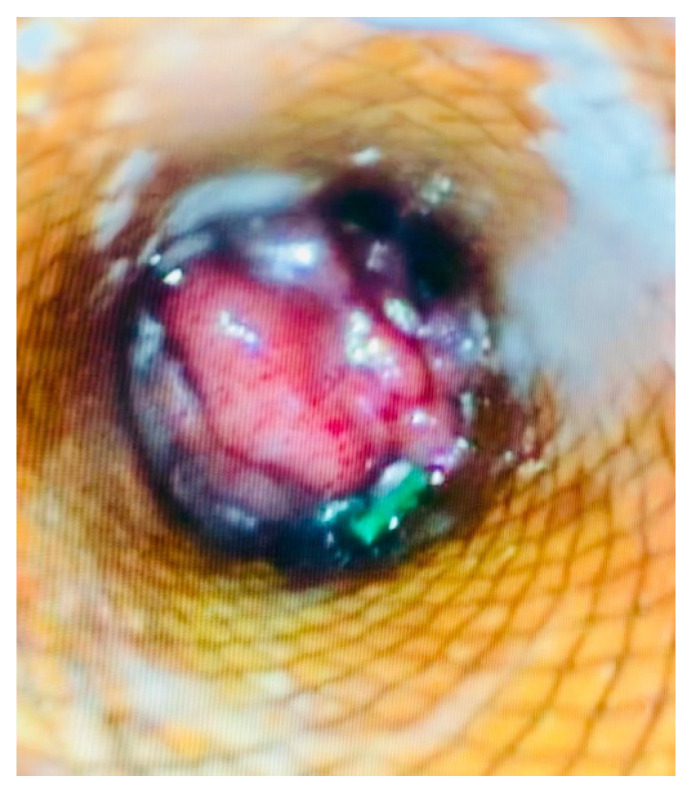
Endoscopic view of jejunal mucosa through the expanded stents ensuring placement.

Proximal and distal landmarks are recognized to appropriately place the stent over the leak site. A guidewire is inserted through the scope, advanced distally to the leak site and immobilized manually by the surgeon through the abdomen (Figure 1). The endoscope is then withdrawn, and the esophageal stent is advanced. It is crucial that sufficient stent length is available above and below the leak site in order to sufficiently seal the anastomotic defect. The endoscope is reinserted parallel to the stent, confirming accurate stent release based on the predetermined landmarks. The stent is gradually deployed, its position is continuously evaluated both endoscopically and manually by the surgeon, and any required minor position adjustments are made. Most importantly, following appropriate placement, the stent is secured to the jejunal loop with one or two interrupted full-thickness absorbable sutures (2-0 or 3-0 Vicryl) through the bowel and stent wall in order to prevent its migration (Figure 2). Additionally, through the scope clips may be placed on the proximal end of the stent according to the endoscopist discretion (Figure 3). The abdominal cavity is then reexamined, washed, and drained appropriately and the abdominal wall is either closed in standard fashion or temporarily depending on the patient status and the possible need for second-look laparotomy. The patient is then transferred to the Intensive Care Unit (ICU) for continued monitoring and supportive care.

In the proposed rendezvous technique, stents are intended to stay in situ for a maximum of 6 weeks in order to avoid embedded stents. Their efficacy in sealing the anastomotic defect is evaluated using imaging studies, such as oral contrast swallow tests or CT scan, depending on the clinical status. Oral feeding is initiated at least 48 h after reoperation. Drain output is carefully monitored and examined in order to confirm sufficient anastomotic leak control. Oral feeding is gradually escalated, providing that patient status allows for it. Patients may be discharged with stent in place, depending on their clinical status and the need for prolonged parenteral antibiotic treatment. Routine endoscopic evaluation is scheduled 6 weeks after stent placement. In case of endoscopic proof of successful healing of the anastomotic leak, the stent is removed endoscopically, since the absorbable have been degraded. A final normal swallow study allows for full oral intake and resumption of adjuvant treatment.

### 2.2. Postoperative Management

In the proposed rendezvous strategy, esophageal stents were intended to remain in situ for a maximum duration of six weeks in order to minimize the risk of stent embedding and facilitate safe endoscopic removal. Postoperative monitoring focused on clinical, laboratory, and radiological parameters indicative of leak control and sepsis resolution. Stent efficacy was assessed using imaging modalities tailored to each patient’s clinical condition. These included oral contrast swallow studies and contrast-enhanced CT scans, which were used to evaluate stent position, confirm adequate sealing of the anastomotic defect, and assess resolution of intra-abdominal collections. Drain output was closely monitored, with both volume and content evaluated to detect ongoing leakage.

Oral feeding was initiated no earlier than 48 h after reoperation, provided that imaging studies demonstrated effective contrast passage without extravasation. Nutritional intake was advanced gradually, beginning with liquids and progressing to soft and regular diets as tolerated. Patients who required prolonged parenteral antibiotic therapy or ongoing supportive care were discharged with the stent in place when clinically appropriate.

Routine endoscopic reassessment was scheduled for approximately six weeks following stent placement. In cases where endoscopic evaluation confirmed complete healing of the anastomotic defect, stent removal was performed endoscopically without complications. Final confirmation of anastomotic integrity was obtained with an oral contrast-enhanced CT scan prior to resumption of full oral intake and, when applicable, adjuvant oncologic therapy.

## 3. Results

### Preliminary Clinical Outcomes

The study population consisted of six patients who developed esophagojejunal anastomotic leakage complicated by sepsis and multiorgan failure. Five patients underwent elective total gastrectomy with D2 lymphadenectomy and Roux-en-Y reconstruction for gastric malignancy with curative intent. One patient underwent urgent gastrectomy for a giant bleeding antral ulcer with high suspicion of malignancy. The majority of patients were male, and the mean age was 79 years, reflecting a frail and high-risk population (Table 1).

All patients developed radiologically confirmed anastomotic leakage within the first 15 postoperative days. Clinical deterioration necessitated ICU admission in all cases, with patients exhibiting hemodynamic instability, respiratory compromise, and biochemical evidence of multiorgan dysfunction. Significant intra-abdominal and pleural effusions were present in all patients at the time of reoperation.

Following relaparotomy and rendezvous stent placement, clinical improvement was observed in five of six patients withing the first 48 h after reoperation. These patients demonstrated progressive normalization of septic parameters, reduced drain output, and radiologic resolution of intra-abdominal collections. One patient exhibited persistent intra-abdominal and pleural effusions with ongoing signs of sepsis, necessitating an additional reoperation. During re-exploration, appropriate stent positioning was confirmed, and further drainage resulted in subsequent clinical improvement.

Thirty-day survival was achieved in five patients (83.3%). One patient required placement of an additional stent due to inadequate initial stent diameter; following correction, the postoperative course was uneventful. Mean time to initiation of oral feeding was 4.5 days following reoperation. The average duration of ICU stay was 3.66 days (Table 2).

Overall, 30-day mortality was 16.67% (1/6). (Table 3) The single death occurred despite radiologically confirmed sealing of the anastomotic defect and was attributed to decompensation of pre-existing comorbidities rather than failure of the stenting strategy. Regarding long-term patient outcomes, two out of six patients (33.3%) are alive after a mean follow-up period of 22.5 months, with the majority of deaths being attributable to malignant disease progression.

Stents remained in situ for a mean duration of 5.2 weeks. Endoscopic stent removal was successfully performed in all surviving patients. Complete healing of the anastomotic defect was confirmed both endoscopically and radiologically in all five patients, allowing safe progression to full oral intake and continuation of planned adjuvant treatment.

## 4. Discussion

Anastomotic leakage after upper GI surgery is a significant complication associated with major morbidity and mortality, even if tackled in the early stages [2]. Despite modern imaging and endoscopic advances that allow for early diagnosis and treatment of an anastomotic defect, the majority of patients face major morbidity with prolonged hospitalization that may delay any required adjuvant therapies [2,3,4,5]. In our case series, all patients had an extended hospitalization period. Additionally, four out of five patients who required adjuvant therapy failed to receive it due to their frail post-discharge status.

Anastomotic leak management remains challenging, due to the variability of clinical presentation, severity, and patient status. Treatment choice ranges from conservative management to endoscopic procedures and operative management. Among endoscopic options, clip-based closure with over-the-scope clips (OTSC) have demonstrated clinical success rates ranging from approximately 66% to 73% for anastomotic leaks in pooled analyses, with higher efficacy when applied early and in smaller defects [18,19]. Through-the-scope clips (TTSC) may achieve even higher closure rates (~95%) in selected small defects, although evidence is limited outside small case series [20]. Endoscopic suturing systems, such as OverStitch, have increasingly been applied, though clinical success specifically for upper GI anastomotic leaks remains lower, with closure rates reported around 27–28% in the largest available cohort studies, reflecting technical complexity and tissue conditions often seen in leak settings [21].

Both stenting and vacuum therapy have proven their utility in anastomotic leak cases after upper GI tract surgery as effective endoscopic therapy options [22,23]. Since its introduction, vacuum therapy has gained popularity mainly due to its effectiveness and the relatively short therapy time required [23]. However, the need for repeated endoscopic procedures and oral feeding discontinuation prevent uniform technique acceptance [22,23]. Stenting remains a commonly employed technique after esophagojejunal anastomotic leakage in many high-volume upper GI centers [22,23,24]. It offers immediate control of intrabdominal spillage and often proves effective as the only measure ensuring proper anastomotic healing [22,23,24]. Especially the use of Fully Covered Self-Expandable Metal Stents (FC-SEMS) has proven to be equally effective to vacuum therapy with comparable therapy results regarding therapy time and associated complications [22]. The combination of stenting with negative pressure therapy through the VacStent system aims to combine advantages of both modalities with promising initial results in esophagogastric anastomotic leak patients [25]. Future implementation in esophagojejunal anastomotic leak patients will demonstrate the technique’s contribution in this patient group. In our series, patient status indicated urgent surgical operation. Stenting was utilized in our patient series as the ideal endoscopic technique to be combined with relaparotomy in our hybrid approach. Technical success was observed in all cases with immediate clinical and imaging resolution of the leak, which translated into favorable survival outcomes.

Stent migration remains a major issue of upper gastrointestinal stents as it can cause rapid-onset relapse of septic profile and may even require reoperation in an already hostile surgical environment [26,27,28,29]. Stent dislodgement is the main complication of their use [29]. In our series, intraoperative stent placement and anchoring proved effective for accurate placement and fixation in all cases, as stent migration was not observed in any case. One patient required repeat stent placement owing to unsuitable stent width. Due to the distal stent anchoring, the initial stent was not removed. The second stent was placed using the stent-in-stent technique, with excellent post-intervention results. Full thickness suture anchoring of the stent provided sufficient structural integrity in order to prevent stent migration in all cases. Additionally, the choice of absorbable sutures of stent anchoring allows for unhindered endoscopic stent removal after completion of treatment.

Our technique’s effectiveness translated into favorable outcomes regarding 30-day mortality. Rendezvous stenting was elected as part of a rescue effort in severely ill patients with major comorbidities, who were reoperated for peritoneal lavage in order to minimize the septic load on already severely ill patients. Despite their unfavorable clinical status, five out of six patients achieved 30-day survival. The one death observed in our mini-series was attributed to dysregulation of other severe comorbidities and was not a direct result of anastomotic leakage.

Stenting in combination with reoperation for wash-out remains a cornerstone of treating upper GI anastomotic leak patients, with spillage who develops signs of peritonitis. In our experience, the modified rendezvous stenting technique was highly effective in offering control of the septic source due to reliable and precise placement, in a series of high-risk patients. Further exploitation of this technique will allow for more accurate conclusions to be drawn. The present study, although bearing promising results, is limited by its own nature. Our novel technique needs to be implemented and evaluated in larger patient population in order to determine the actual technique’s benefit.

## 5. Conclusions

Upper GI anastomotic leak is associated with severe sepsis and metabolic derangement of the patient. A multimodal approach is required in order to achieve the optimal outcome, with the goal of locally controlling the septic source while minimizing physiological derangement. Several strategies including primary closure of the defect, EndoVac treatment and endoscopic clipping have been employed in order to contain the anastomotic leak and allow for proper healing. According to our experience, stenting the anastomotic defect remains a useful tool in this effort to minimize peritoneal spillage while offering a scaffold for proper anastomotic healing. The modified rendezvous technique presented in this technical report offers an effective hybrid method in highly demanding cases, which can be implemented with safety and accuracy. Further experience is needed in order to prove the exact place for the technique.

## Figures and Tables

**Table 1:** Patients’ characteristics.

Variable	N = 6
Age, years, Mean (Range)	79 (73–85)
Gender	
Male	4
Female	2
Disease	
Gastric malignancy	5
Benign disease	1
Charlson Comorbidity Index (CCI)	
Stage	
Stage II	4
Stage IV	1
Benign disease	1
ECOG status	
0	1
1	3
2	2

**Table 2:** Perioperative data.

	Mean (Range)
Defect size	1.3 cm (0.5–3)
ICU stay (days)	3.66 days (2–6)
Time to oral feeding (days)	4.5 days (2–9)
Time to anastomotic healing (weeks)	5.2 (3–6)

**Table 3:** Patients’ postoperative outcomes.

Patient	30-Day Survival (Yes/No)	Need for Additional Stent (Yes/No)	Stent in Position (Weeks)	Immediate Clinical Efficacy (Yes/No)
1	Yes	-	6	Yes
2	Yes	Yes	6/6	Yes
3	Yes	-	3	No
4	No	-	1	Yes
5	Yes	-	5	Yes
6	Yes	-	6	Yes

## Data Availability

The original contributions presented in this study are included in the article. Further inquiries can be directed to the corresponding author.

## Abbreviations

The following abbreviations are used in this manuscript:

GI	Gastrointestinal
CT	Computed tomography
ICU	Intensive Care Unit
